# Imatinib-Induced Lichen Planus in Chronic Myeloid Leukemia: A Case Series

**DOI:** 10.7759/cureus.39064

**Published:** 2023-05-15

**Authors:** Anuhya Yelisetti, Nirmala Devi Chandrasekaran, V.M. Durai Mavalavan, Janardhanan Kumar, Hariharan Eswaran

**Affiliations:** 1 General Medicine, SRM Medical College Hospital and Research Centre, Chengalpattu, IND; 2 Medical Oncology, SRM Medical College Hospital and Research Centre, Chennai, IND; 3 General Medicine, SRM Medical College Hospital and Research Centre, Chennai, IND; 4 Internal Medicine, SRM Medical College Hospital and Research Centre, Chennai, IND

**Keywords:** drug reaction, leukemoid reaction, chronic myeloid leukaemia, lichen planus, imatinib mesylate

## Abstract

Chronic myeloid leukemia (CML) is a myeloproliferative disorder in which the Philadelphia chromosome is the cytogenetic hallmark. It is characterized by the t (9;22) translocation, which in turn creates the chimeric BCR-ABL oncogene coding for a constitutively activated tyrosine kinase. Imatinib mesylate is a tyrosine kinase inhibitor that targets the BCR-ABL protein, c-KIT, and platelet-derived growth factor (PDGF) receptors and is used to treat CML, gastrointestinal stromal tumors, and dermato-fibrosarcoma protuberant. The development of the specific inhibitor of BCR-ABL tyrosine kinase has been a notable success and approved as the first-line treatment for CML. Although adverse cutaneous reactions to imatinib mesylate are not infrequent, their clinical and histopathological features have generally been poorly characterized. Here we report three rare cases of cutaneous lichenoid eruptions that occurred during the treatment of CML with imatinib mesylate.

## Introduction

Imatinib, a 2-phenyl amino pyrimidine derivative, is a tyrosine kinase inhibitor that targets BCR-ABL, the abnormal gene product of the Philadelphia chromosome t (9;22). Inhibition of this enzyme mitigates cell proliferation and induces apoptosis in BCR-ABL-positive chronic myeloid leukemia (CML) cells. Imatinib also has activity on platelet-derived growth factor (PDGF), stem cell factor (SCF), c-KIT, and cellular events mediated by PDGF and SCF [1-2].

Drug-induced lichen planus (also known as lichenoid drug reaction) is characterized by flat-topped, polygonal, erythematous, or violaceous papules resembling idiopathic lichen planus. They usually present as psoriasiform lesions and are larger than those associated with idiopathic lichen planus. Cutaneous involvement in lichenoid drug reaction typically affects the lower extremities and the trunk, in a symmetrical distribution [3]. A variety of drugs have been implicated in the etiology of drug-induced lichen planus, including, but not limited to, quinine, quinidine, penicillamine, and dental materials such as mercury and gold [3], and, in rare cases, immunomodulators and newer antineoplastic agents like imatinib mesylate [4].

Lichenoid drug reaction falls under the umbrella of interface dermatitis characterized by common histopathological elements of epidermal basal cell injury and perivascular mononuclear inflammatory cells in the papillary and mid-dermis, consisting of activated T cells, macrophages, and dendritic cells [5]. Histopathological manifestations specific for lichenoid drug reaction include numerous necrotic keratinocytes (sometimes in clusters), focal parakeratosis, as well as plasma cells and eosinophils [6]. This case series reports three cases of imatinib-induced lichenoid drug reactions.

## Case presentation

Case 1

A 67-year-old male presented with complaints of abdominal distension and discomfort for one month. On examination of the abdomen, hepatosplenomegaly was detected. His hemogram revealed a hemoglobin (Hb) level of 11.7 g/dL, a total white blood cell (WBC) count of 2,42,300 cells/cu.mm, and a platelet count of 3,20,000 cells/cu.mm. Peripheral smear showed markedly increased WBCs with left shift, increase in blasts, and basophilia suggestive of CML in the chronic phase. Bone marrow biopsy confirmed CML in the chronic phase, and fluorescence in situ hybridization (FISH) was 100% positive for BCR-ABL translocation on chromosome 22, the Philadelphia chromosome. The patient was started on tablet (T.) imatinib 400 mg once daily, T. allopurinol thrice a day, and multivitamins.

**Figure 1:**
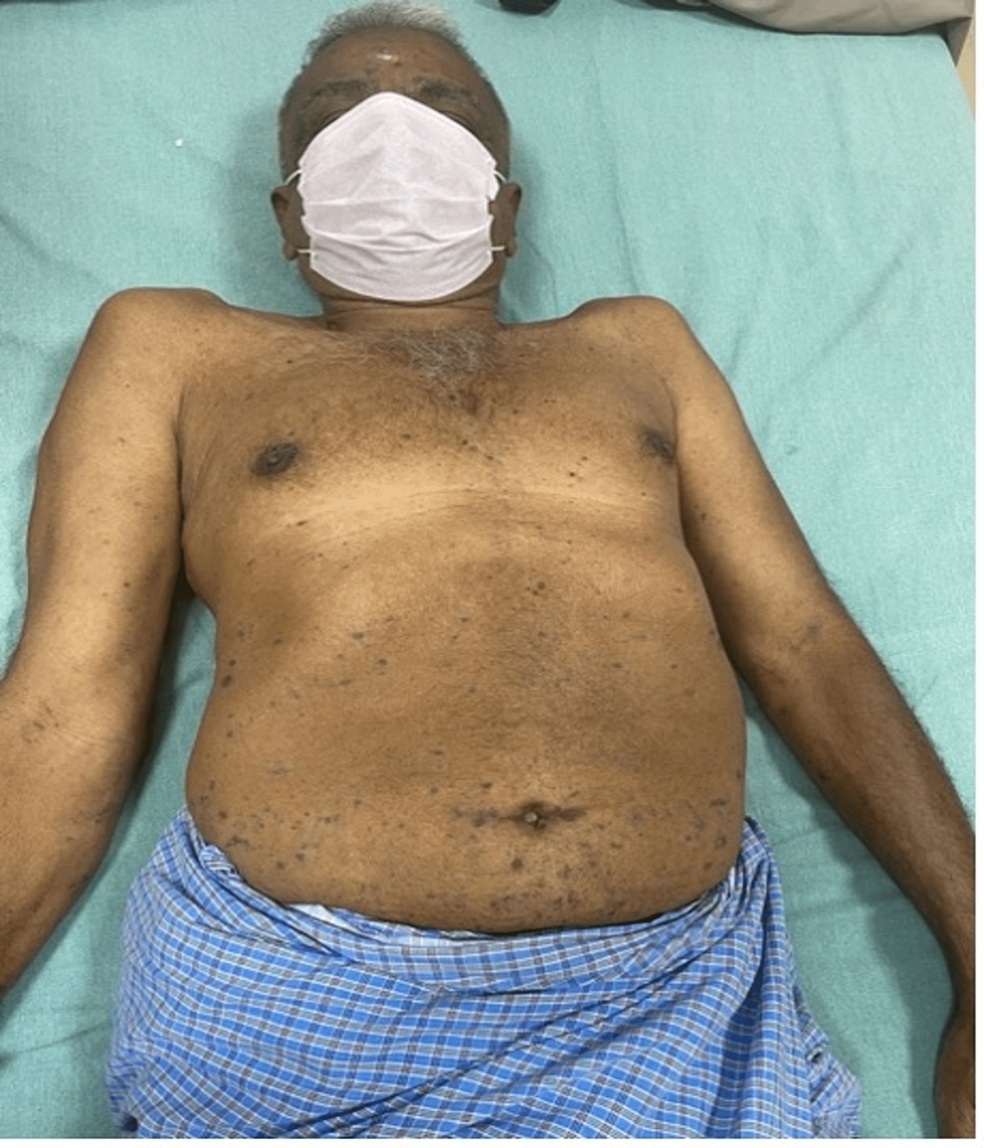
Multiple violaceous flat-topped papules over the trunk

Two months after the initiation of treatment, the patient presented to us with multiple violaceous, scaly, and hyperpigmented patches and a few plaques over the abdomen and trunk, as shown in Figure 1. A skin biopsy was performed, and microscopy showed hyperkeratotic, hyperplastic squamous epithelium, mononuclear infiltration in subepithelial tissue, and disruption of dermo-epidermal junction suggestive of lichen planus. Hemogram at the time showed Hb of 11.2 g/dL, WBC of 28,590 cells/cu.mm, and a platelet count of 3,30,000 cells/cu.mm. Viral serology for HIV, hepatitis B surface antigen (HBsAg), and hepatitis C virus (HCV) was non-reactive. A diagnosis of lichen planus secondary to imatinib was made. As the reaction was mild, the drug was not interrupted, and the patient was treated with topical steroids and antihistamines. At the three-month follow-up, a complete resolution of hyperpigmentation and plaques was observed.

Case 2

A 42-year-old female, known to have diabetes for three years, presented with complaints of increased frequency and burning micturition for five days. On routine evaluation, the hemogram showed Hb of 10.3 g/dL and WBC of 1,21,760 cells/cc.mm with neutrophils (N)-78, lymphocytes (L)-5, and basophils (B)-8. Peripheral smear revealed increased total counts with left shift, abnormal nuclear-cytoplasmic ratio, and basophilia. Viral serology tested negative. Bone marrow aspiration showed CML in an accelerated phase. FISH for Philadelphia chromosome (9;22) was positive for ABL-BCR translocation. Treatment included intravenous (IV) ceftriaxone 1 gram twice a day for urinary tract infection (UTI), T. metformin 50 mg with vildagliptin 500 mg twice daily for diabetes, and the patient was started on T. imatinib 400 mg once daily and capsule Neurobion Forte at discharge.

**Figure 2:**
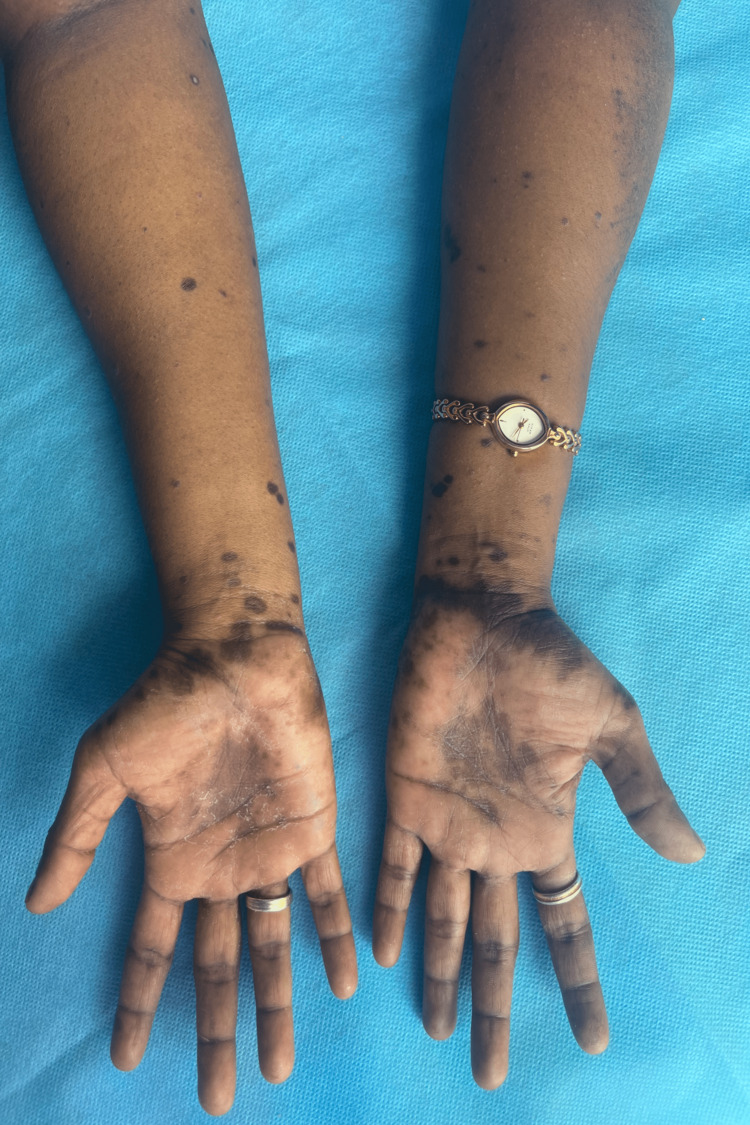
Multiple hyperpigmented, scaly patches and a few plaques noted over both hands and forearms

Six weeks later, the patient presented with multiple pruritic violaceous lesions over the buccal mucosa, lower gingiva, labia, and bilateral forearms (Figure 2), suggestive of lichen planus or lichenoid eruption. Biopsy of the lesion revealed degeneration of the basal cell layer, hyperkeratosis, thickening of the granular cell layer, and band-like infiltration of the sub-epidermal layer, which is suggestive of a lichenoid drug reaction. A diagnosis of imatinib-induced lichen planus was reached. As the patient had a burning sensation in the oral cavity, the drug was withheld for two weeks, and she was managed with oral and topical steroids as well as antihistamines. Once the lesions started resolving, imatinib was restarted in the third week, and oral steroids were tapered and eventually stopped. The patient showed complete resolution of the lesions by the sixth month.

Case 3

A 56-year-old male with no prior comorbidities presented with abdominal discomfort and easy fatiguability for the past three months. On examination, severe pallor was present, and massive splenomegaly was detected. Hemogram revealed Hb levels of 8.9 g/dL, WBC of 78,300 cells/cu.mm, and platelet count of 3,57,200 cells/cu.mm. Viral serology for HIV, HbsAg, and HCV was negative. Peripheral smear revealed abundant leucocytes with left shift, basophilia, and blast cells, suggestive of CML in the chronic stage. FISH for Philadelphia chromosome (9;22) was positive for ABL-BCR translocation. Bone marrow biopsy also confirmed the diagnosis of CML. The patient was started on T. imatinib 400 mg once a day and T. allopurinol thrice daily.

**Figure 3:**
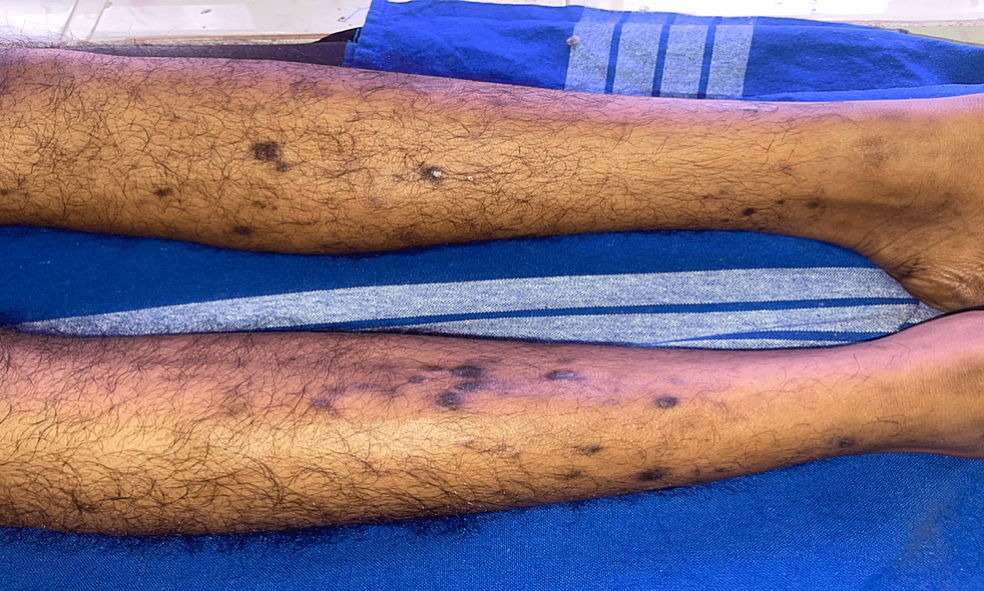
Numerous violaceous raised papules noted over both legs

One month after the initiation of treatment, the patient developed multiple scaly, flat-topped papules and painless plaques over the anterior aspect of both lower limbs and the trunk region (Figure 3). Over the next few days, the lesions worsened, and the patient developed itching and redness. Suspecting imatinib-induced skin reaction, the drug was withheld, and a skin biopsy was performed. Skin biopsy from the trunk revealed hypergranulosis, hyperkeratosis, and basal cell vacuolization. The dermis showed band-like moderate lymphocytic inflammatory cell infiltrate, predominantly perivascular, suggestive of lichen planus. The patient was treated with topical and oral corticosteroids, antihistamines, and local emollients. After about three weeks of treatment, the lesions improved, and imatinib was restarted.

## Discussion

Lichen planus is a papulosquamous eruption characterized by flat-topped, violaceous papules involving the skin and mucosal surfaces; individual papules may coalesce to form plaques. Lichenoid drug reactions may manifest with features typical of lichen planus or can be atypical. Lichenoid drug eruption lesions are symmetric eczematous papules with marked desquamation and are generally larger than typical idiopathic lichen planus lesions. They involve the oral mucosa less often and are largely devoid of Wickham striae, and residual hyperpigmentation is common. The occurrence of these lichenoid drug reaction lesions is usually dose-dependent [3,7]. Non-lichenoid dermatoses associated with imatinib therapy may include maculopapular eruption, edema, Stevens-Johnson syndrome, toxic epidermal necrolysis, acute generalized exanthematous pustulosis, vasculitis, and mycosis fungoides-like eruption [8].

Imatinib is a competitive inhibitor of BCR-ABL tyrosine kinase resulting in cell cycle regulation, apoptosis of tumorigenic cells, and resolution of gene stability. It is the first-line treatment in patients with newly diagnosed Philadelphia chromosome-positive CML at a dose of 400 mg. Higher doses are recommended in accelerated or myeloid blastic phases or if an adequate hematologic or cytogenetic response is not achieved. Lichenoid reactions have been reported at these therapeutic doses and seem to occur with greater frequency at higher doses [1,9-11].

The patient from the first reported case had milder symptoms and was maintained at the usual dose of 400 mg once daily with a topical steroid added. The other two had comparatively more severe symptoms, and in addition to topical steroids, both required interruption in imatinib treatment. All cases showed complete resolution of lesions with symptomatic improvement. A photo-distributive pattern of lesions has been described in most of the reported cases of idiopathic lichen planus [12], but the three cases in this study did not follow any such pattern. Though a photo aggravation response was seen in the first male patient who was a farmer by occupation, topical steroids and advice to avoid sunlight led to the early resolution of lesions. In case 3, the patient also had generalized lightening of skin color and cicatricial scarring type of alopecia that was eventually controlled by topical and oral steroids. The condition mimicked seborrheic dermatitis and resolved after withholding imatinib. There was complete resolution of lesions with hair regrowth.

A bilateral presentation of lesions is more idiopathic than drug-induced. The mechanism of imatinib-induced skin lesions remains elusive. Due to its low molecular weight, imatinib is not likely to be immunogenic. The association between the coexistence of hepatitis C and the propensity to develop imatinib-induced lichen planus was also considered. All three cases were subjected to viral markers but tested negative. To date, there is no valid hypothesis to support the co-occurrence of hepatitis C and lichen planus in a patient’s presentation [13]. Only a few reports of imatinib-associated lichenoid drug eruption have been described over the last decade. An analysis done in 2013 showed only 16 previously reported cases of lichen planus secondary to imatinib in CML patients [14].

## Conclusions

Our case series highlighted the potential of imatinib to cause lichen planus and the importance of differentiating this entity from its idiopathic counterpart. Lichen planus is a rather rare manifestation of imatinib use, with very few documented case reports available for reference. None of the patients ever treated with imatinib for CML had any deleterious severe lichenoid reaction that would sabotage the principal treatment in due course. Topical and oral steroids play a pivotal role in bridging the cutaneous interactions caused by imatinib in CML patients. Furthermore, early recognition of the morphological pattern of such a drug eruption is crucial to prevent the subsequent discontinuation of imatinib, which has radically transformed the treatment and prognosis of CML.
